# Pharmacological Inhibition of FAK-Pyk2 Pathway Protects Against Organ Damage and Prolongs the Survival of Septic Mice

**DOI:** 10.3389/fimmu.2022.837180

**Published:** 2022-02-01

**Authors:** Gustavo Ferreira Alves, Eleonora Aimaretti, Giacomo Einaudi, Raffaella Mastrocola, Junior Garcia de Oliveira, Debora Collotta, Elisa Porchietto, Manuela Aragno, Carlo Cifani, Regina Sordi, Christoph Thiemermann, Daniel Fernandes, Massimo Collino

**Affiliations:** ^1^ Department of Neurosciences (Rita Levi Montalcini), University of Turin, Turin, Italy; ^2^ Department of Pharmacology, Federal University of Santa Catarina, Florianópolis, Brazil; ^3^ Department of Clinical and Biological Sciences, University of Turin, Turin, Italy; ^4^ Pharmacology Unit, School of Pharmacy, University of Camerino, Camerino, Italy; ^5^ William Harvey Research Institute, Bart’s and The London School of Medicine and Dentistry, Queen Mary University of London, London, United Kingdom

**Keywords:** sepsis, NLRP3 inflammasome, inflammation, Focal adhesion kinase (FAK), proline-rich tyrosine kinase 2 (PYK2)

## Abstract

Sepsis and septic shock are associated with high mortality and are considered one of the major public health concerns. The onset of sepsis is known as a hyper-inflammatory state that contributes to organ failure and mortality. Recent findings suggest a potential role of two non-receptor protein tyrosine kinases, namely Focal adhesion kinase (FAK) and Proline-rich tyrosine kinase 2 (Pyk2), in the inflammation associated with endometriosis, cancer, atherosclerosis and asthma. Here we investigate the role of FAK-Pyk2 in the pathogenesis of sepsis and the potential beneficial effects of the pharmacological modulation of this pathway by administering the potent reversible dual inhibitor of FAK and Pyk2, PF562271 (PF271) in a murine model of cecal ligation and puncture (CLP)-induced sepsis. Five-month-old male C57BL/6 mice underwent CLP or Sham surgery and one hour after the surgical procedure, mice were randomly assigned to receive PF271 (25 mg/kg, s.c.) or vehicle. Twenty-four hours after surgery, organs and plasma were collected for analyses. In another group of mice, survival rate was assessed every 12 h over the subsequent 5 days. Experimental sepsis led to a systemic cytokine storm resulting in the formation of excessive amounts of both pro-inflammatory cytokines (TNF-α, IL-1β, IL-17 and IL-6) and the anti-inflammatory cytokine IL-10. The systemic inflammatory response was accompanied by high plasma levels of ALT, AST (liver injury), creatinine, (renal dysfunction) and lactate, as well as a high, clinical severity score. All parameters were attenuated following PF271 administration. Experimental sepsis induced an overactivation of FAK and Pyk2 in liver and kidney, which was associated to p38 MAPK activation, leading to increased expression/activation of several pro-inflammatory markers, including the NLRP3 inflammasome complex, the adhesion molecules ICAM-1, VCAM-1 and E-selectin and the enzyme NOS-2 and myeloperoxidase. Treatment with PF271 inhibited FAK-Pyk2 activation, thus blunting the inflammatory abnormalities orchestrated by sepsis. Finally, PF271 significantly prolonged the survival of mice subjected to CLP-sepsis. Taken together, our data show for the first time that the FAK-Pyk2 pathway contributes to sepsis-induced inflammation and organ injury/dysfunction and that the pharmacological modulation of this pathway may represents a new strategy for the treatment of sepsis.

## Introduction

Sepsis is a major public health concern, responsible for high mortality and morbidity rates, followed by a reduced quality of life for survivors (1–3). The latest data on the global sepsis burden reported 48.9 million incident cases and 11.0 million sepsis-related deaths, representing 19.7% of all deaths worldwide (4). Despite the progress in clinical and basic research, the prognosis of septic patients remains remarkably poor, prompting the World Health Organization and World Health Assembly to recognize sepsis as a global health priority and, thus, adopting a resolution to improve the prevention, diagnosis and management of sepsis (5). The morbidity and mortality in sepsis are driven by organ dysfunction (6) and, among the multitude of triggering mechanisms, acute hyperinflammation has a substantial impact on host responses, which in turn contributes to sepsis-related multiple organ failure (MOF) (7). Very recently, two non-receptor proteins tyrosine kinase, belonging to the Focal adhesion kinase (FAK) family, namely FAK and Proline-rich tyrosine kinase 2 (Pyk2), have been identified as new key players in mediating the inflammatory response involved in the pathogenesis of endometriosis (8), atherosclerosis (9) and asthma (10) as well as in tumorigenesis and metastasis formation (11, 12). FAK and Pyk2 are ubiquitously expressed and they share the same three-domain organization, with two non-catalytic domains, the band 4.1, ezrin, radixin and moesin (FERM) domain and the focal adhesion targeting (FAT) domain, linked by a central kinase domain (13). In addition to the kinase function, both FAK and Pyk2 act as scaffold proteins and play a crucial role in downstream integrin signaling (14, 15). Despite their homology, the signaling events that lead to activation of these two kinases differ. FAK is mainly activated by integrins, growth factor receptors and cytokine receptors, leading to overproduction of several pro-inflammatory mediators and cytokines (16). In contrast, Pyk2 has the exclusive ability to sense calcium ions and it can be overactivated under lipopolysaccharide (LPS) stimulation resulting in overproduction of chemokines that regulate migration and infiltration of monocytes/macrophages, including monocyte chemotactic protein-1 (MCP-1), through a p38-MAPK pathway dependent mechanism (17, 18). Besides, a compensatory upregulation in Pyk2 levels has been documented following pharmacological or genetic inhibition of FAK (19, 20). Thus, the selective and simultaneous inhibition of both proteins may represent an innovative pharmacological approach to counteract FAK-Pyk2-mediated inflammatory response and recently published papers have demonstrated the efficacy of a FAK-Pyk2 dual inhibitor, PF562271 (PF271), to counteract leukocyte infiltration and vascular inflammation in both *in vitro* and *in vivo* conditions (21, 22). Despite few studies have shown that LPS-induced endotoxemia evokes FAK activation, which contributes to exacerbate the inflammatory response, leading to organ damage and increased mortality (23, 24), so far, no experimental data have been reported on the potential effects of pharmacological modulation of the FAK-Pyk2 signaling pathway against sepsis. Thus, the present study was designed to address this issue by investigating the potential beneficial effects of a potent reversible dual inhibitor of both FAK and Pyk2 (PF271) in a murine model of polymicrobial sepsis.

PF271 is an ATP-competitive, reversible inhibitor for both FAK and Pyk2 with IC_50_ of 1.5 nmol/L and 13 nmol/L, respectively (25), which has completed a phase I clinical trial (NCT00666926) in patients with advanced solid tumors (26, 27).

## Material and Methods

### Animals and Ethical Statement

Male, five-month-old C57BL/6 mice (from Envigo laboratories, IT), weighing 30-35 g were used and kept under standard laboratory conditions. The animals were housed in an environment with temperature (25 ± 2°C) and light/dark cycle (12/12 h) automatically controlled, as well as *ad libitum* access to food and water. The animals were kept under standard laboratory conditions for four weeks before the start of the experimental procedures. The animal protocols are reported in compliance with the ARRIVE guidelines (28) and the MQTiPSS recommendations for preclinical sepsis studies (29) and they were approved by the local Animal Use and Care Committees as well as the National Authorities (Ethical number approvals: 420/2016-PR, Italy and 7936220321 Brazil).

### Polymicrobial Sepsis

Sepsis was induced through the cecal ligation and puncture (CLP) introduced by Wichterman and co-workers (1980) (30) with slight modifications. Mice were anesthetized with 3% isoflurane (IsoFlo, Abbott Laboratories) together with 0.4 L/min oxygen in an anaesthesia chamber. Once mice were sedated, they were kept anesthetized by administration of 2% isoflurane (via nosecone) together with 0.4 L/min oxygen throughout the surgical procedure. Through a thermal blanket, the body temperature was maintained at 37°C and constantly monitored using a rectal thermometer. Mice were submitted to a mid-line laparotomy of approximately 1.0 cm and, after location and exposure, the cecum was ligated with a cotton thread right below the ileocecal valve. A single through-and-through puncture of the cecum was carried out with a sterile 21 G needle and the cecum was lightly compressed to leak a small amount (droplet) of feces. Then, the cecum was carefully relocated into peritoneal cavity, which was sutured with silk thread. Sham-operated mice underwent the same procedure, but without CLP. Immediately after the surgical procedures, each mouse received an analgesic agent (Carprofen, 5 mg/kg, s.c.) and resuscitation fluid (37°C, 0.9% NaCl, 50 mL/kg, s.c.) in order to support the hemodynamic situation of the animals and to induce a hyperdynamic shock phase (31, 32). Mice were left on a homeothermic blanket, being constantly monitored until they recover from anesthesia and then placed back into fresh clean cages.

Twenty-four hours post-CLP, the body temperature was recorded, and a blinded assessment of a clinical score was performed to evaluate the symptoms consistent with murine sepsis. The 6 following signs were used to score the health of experimental mice: lethargy, piloerection, tremors, periorbital exudates, respiratory distress, and diarrhea. An observed clinical score >3 was defined as developing severe sepsis, while a score ≤3 indicated the development of moderate sepsis (33). Details on the clinical score are reported as supplementary material.

### Drug Treatment

Twenty-five mice were randomly distributed into three groups and subjected to either Sham or CLP procedure: Sham (Vehicle), CLP (Vehicle) and CLP+PF271. One hour after surgeries, mice received once either Vehicle (5 µl/g) (5% DMSO; 40% PEG 300; 5% Tween 80; 50% ddH2O) or PF562271 (PF271) (#PF562271, Selleck Chemicals, Sylvanfield Dr, Houston, USA) subcutaneously at the dose of 25 mg/kg, based on previous *in vivo* studies (21, 34).

### Survival Study

To evaluate the potential therapeutic effect of PF271 on sepsis, 28 mice were subjected to CLP-induced sepsis and then randomly allocated into two groups, receiving either Vehicle or PF271 (25 mg/kg) once subcutaneously, one hour after CLP; n = 14 for each group. Survival rates were assessed every 12 h over the subsequent 5 days.

### Plasma and Organ Collection

Twenty-four hours after surgery, mice were anesthetized using isoflurane and euthanized by cardiac exsanguination. Blood samples were collected in microtubes containing EDTA (17.1 µM/mL of blood) and then centrifuged at 13,000 *g* at R.T. to obtain plasma content. Liver and kidney samples were harvested and conserved in cryotubes containing or not optimal cutting temperature (OCT) compound and frozen in liquid nitrogen. Samples were stored at -80°C until analysis, which were performed blindly.

### Biochemical Analysis

Plasma levels of aspartate aminotransferase (AST) (#7036), Alanine aminotransferase (ALT) (#7018), creatinine (#7075), urea (#7144) and lactate (#7170) were measured using commercially available clinical assay kits (FAR Diagnostics, Verona, Italy) following the manufacturer’s instructions.

### Cytokine and Hormone Analysis

Cytokines were determined in plasma by using the Luminex suspension bead-based multiplexed Bio-Plex Pro™ Mouse Cytokine Th17 Panel A 6-Plex (#M6000007NY) assay (Bio-Rad, Kabelsketal, Germany). The cytokines IFN-γ, IL-1β, IL-6, IL-10, IL-17 and TNF-α were measured according to the manufacturer’s instructions. Plasma hormone levels of PAI-1 (#ab197752) and Resistin (#ab205574) were measured through conventional enzyme-linked immunosorbent assays (ELISA) according to the manufacturer’s instructions.

### Myeloperoxidase (MPO) Activity Assay

MPO activity assay has been described previously (35). About 100 mg of liver and kidney samples were homogenized, centrifuged and assayed for MPO activity by measuring the H_2_O_2_-dependent oxidation of 3,3′,5,5′-tetramethylbenzidine (TMB). MPO activity was expressed as optical density (O.D.) at 650 nm per mg of protein.

### Immunohistochemistry

MPO and NOS-2 immunopositivity were analyzed by immunohistochemistry on 10 µm frozen tissue sections of liver and kidney samples. Slides were fixed (10 min) in acetone post-sectioning. Sections were incubated over 2 h with primary antibodies at room temperature (MPO, Abcam, #ab9535, dilution 1:25; iNOS, Santa Cruz, #sc-651, dilution 1:50) and subsequently for 1 h with horseradish peroxidase (HRP)-conjugated secondary antibodies (dilution 1:200). The chromogenic detection was obtained by adding 3,3’-diaminobenzidine (DAB) substrate (2 min of exposition). Finally, the nucleus was counterstained with hematoxylin (10 min).

### qRT-PCR

Total RNA was extracted from the liver (15 mg) and kidney (17 mg) using Animal Tissue RNA Purification Kit (#25700 Norgen Biotek Corporation, Thorold, ON, Canada) according to manufacturer’s instructions. The RNA concentration and purity were measured using a nanodrop (Titertek-Berthold) machine and 200 ng/µL (liver) or 120 ng/µL (kidney) were reverse transcripted. cDNA was prepared using the SensiFAST™ cDNA Synthesis Kit 50 reactions (#BIO-65053 Meridian Bioscience, USA) according to manufacturer’s instructions. Real time qPCR was performed using 100 ng of cDNA (liver and kidney) through the SensiFAST™ SYBR^®^ No-ROX Kit (#BIO-98005 Meridian Bioscience, USA) according to manufacturer’s instructions. PCR reaction was carried out on the CFX Connect Real-Time PCR Detection System (Bio-Rad). Relative gene expression was obtained after normalization to housekeeping genes (β-actin and GAPDH) using the formula 2^-ΔΔCT^ as previously described (36) and folds change was determined by comparison to Sham group. The QuantiTect primers β-actin (#QT00095242, Mm_Actb_1_SG), GAPDH (#QT01658692, Mm_Gapdh_3_SG), ICAM1 (#QT00155078, Mm_Icam1_1_SG), VCAM1 (#QT00128793, Mm_Vcam1_1_SG), E-Selectin (#QT00114338, Mm_Sele_1_SG) used in this study were purchased from QIAGEN (Germantown, MD, EUA).

### Western Blot Analysis

About 50 mg of liver and kidney samples were homogenized and centrifuged (13,000 *g*, 10 min, 4°C). Supernatants were collected and the protein content was determined using a BCA protein assay following the manufacturer’s instructions. 50 µg of total proteins were loaded for immunoblot experiments. Proteins were separated by either 8% or 10% sodium dodecyl sulphate-polyacrylamide gel electrophoresis (SDS-PAGE) and transferred to a polyvinylidene difluoride (PVDF) membrane, which was then incubated with primary antibodies (dilution 1:1000). The antibodies used were: rabbit anti-Thr^180^/anti-Tyr^182^ p38 (Cell Signaling #9211); rabbit anti-total p38 (Cell Signaling #9212); mouse anti-NRLP3 (Adipogen- AG-20B-0014-C100); rabbit anti-Caspase-1 (Cell Signaling #24232); rabbit anti-Tyr^397^ FAK (Cell Signaling #3283); rabbit anti-total FAK (Cell Signaling #3285); mouse anti-Tyr^402^ PyK2 (Cell Signaling #3291); mouse anti-total PyK2 (Santa Cruz #sc-393181); rabbit anti-β-actin (Cell Signaling #4970). Blots were then incubated with a secondary antibody (Cell Signaling - anti-mouse #7076; anti-rabbit #7074) conjugated with HRP (dilution 1:10000) and developed using the ECL detection system. The immunoreactive bands were analyzed by the Bio-Rad Image Lab SoftwareTM 6.0.1 and results were normalized to sham.

### Statistical Analysis

Power analysis for the study design through G-Power 3.1™ software (37). Data are expressed as dot plots for each animal and as mean ± SD of 5-10 mice. The standard distribution of data was verified by Shapiro-Wilk normality test and the homogeneity of variances by Bartlett test. The statistical analysis was performed by one-way ANOVA, followed by Bonferroni’s *post-hoc* test. Data that were not normally distributed, non-parametric statistical analysis was applied through Kruskal-Wallis followed by Dunn’s *post hoc*-test. Differences in the survival study were determined with a log-rank (Mantel-Cox) test. A P value <0.05 was considered significant. Statistical analysis was performed using GraphPad Prism^®^ software version 7.05 (San Diego, California, USA).

### Materials

Unless otherwise stated, all reagents were purchased from the Sigma-Aldrich Company Ltd. (St. Louis, Missouri, USA). The BCA Protein Assay kit was from Pierce Biotechnology Inc. (Rockford, IL, USA). Antibodies were from Cell-Signaling Technology (Beverly, MA, USA).

## Results

### PF271 Improves Severity Score and Prolongs Survival of Septic Mice

**Figure 1:**
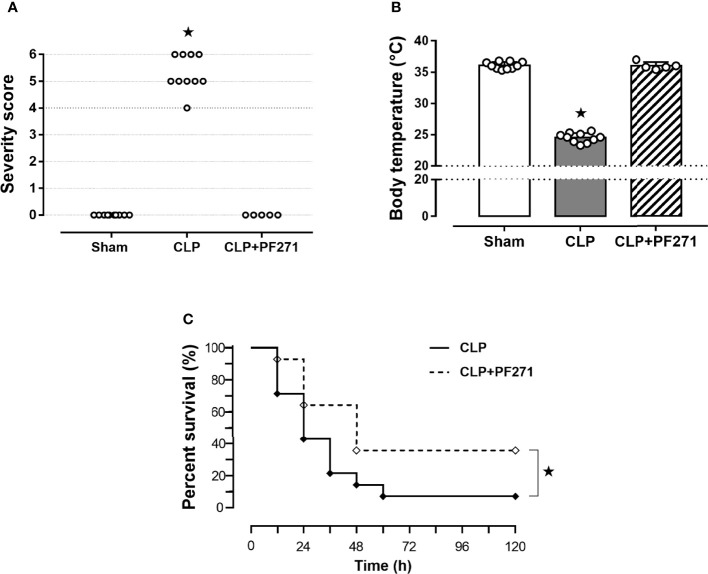
Effect of PF271 on severity and survival in experimental sepsis. Mice were randomly selected to undergo Sham or CLP surgery. One hour later, CLP mice were treated once with either Vehicle or PF271 (25 mg/kg s.c.). Twenty-four hours after Sham or CLP procedure, severity score **(A)** and body temperature **(B)** were recorded. Data are expressed as dot plots for each animal and as mean ± SD of 5-10 mice per group. The Kruskal-Wallis followed by Dunn’s *post hoc*-test was applied to assess the severity score, whereas for body temperature, one-way ANOVA followed by Bonferroni’s *post hoc*-test was used. *p < 0.05 CLP *vs* Sham/CLP+PF271. The mortality rate was recorded over a 5-day period **(C)**. A log-rank test was used for the comparison of the survival curves (n = 14 mice per group). *p < 0.05 CLP *vs* CLP+PF271.

Mice that underwent CLP surgery developed clinical signals of severe sepsis (score >3) (**Figure 1A**) associated with hypothermia (24.51 ± 0.24°C) at 24 h after the onset of experimental sepsis (**Figure 1B**). Interestingly, treatment with PF271 demonstrated a protective effect in septic mice, as the severity score (score=0) and body temperature (36.0 ± 0.29°C) did not differ (P>0.05) from those observed in the Sham group (**Figures 1A, B**). To further determine the overall long-term effect of PF271 treatment, we performed a survival study in septic mice. As shown in **Figure 1C**, the median survival was 24 h in the CLP-mice treated with vehicle and 48 h in the CLP-mice treated with PF271. PF271 significantly prolonged the survival time of septic mice since 93% of the CLP+Vehicle mice died within 120 h, while the CLP-mice treated with PF271 had significantly reduced mortality of 64% (hazard ratio: 0.33; 95% confidence interval: 0.12–0.89; P < 0.05).

### PF271 Administration Reduces Plasma Levels of Biomarkers of Organ Damage in CLP-Induced Sepsis

**Figure 2:**
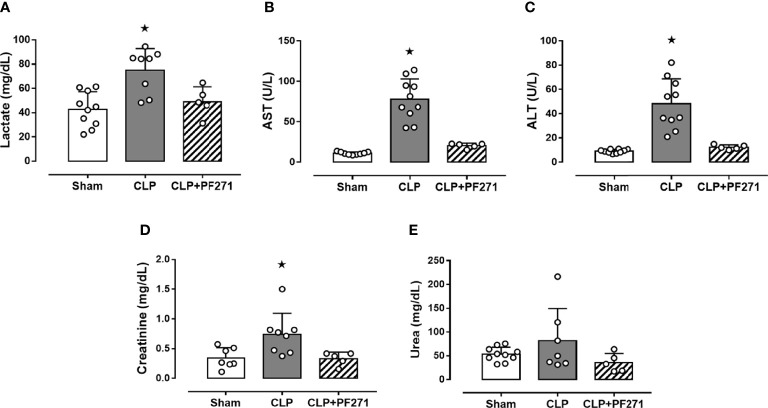
Effect of PF271 on plasma CLP-induced organ damage markers. Mice were randomly selected to undergo Sham or CLP surgery. One hour later, CLP mice were treated once with either vehicle or PF271 (25 mg/kg s.c.). Twenty-four hours after Sham or CLP procedure, the animals’ blood was collected. Plasma levels of lactate **(A)**, aspartate transaminase (AST) **(B)**, alanine transaminase (ALT) **(C)**, creatinine **(D)** and urea **(E)** were determined. Data are expressed as dot plots for each animal and as mean ± SD of 5-10 mice per group. Statistical analysis was performed by one-way ANOVA followed by Bonferroni’s *post hoc* test. *p < 0.05 CLP *vs* Sham/CLP+PF271.

CLP-mice treated with vehicle exhibited a significant increase in their plasma levels of lactate (74.91 ± 6.31 mg/dL), AST (77.5 ± 8.02 U/L), ALT (48.19 ± 6.47 U/L) and creatinine (0.74 ± 0.13 mg/dL) compared to Sham animals (42.86 ± 4.55; 11.0 ± 0.57; 9.09 ± 0.48; 0.34 ± 0.06, respectively), hence, suggesting poor tissue perfusion (lactate), hepatic (AST and ALT) and renal (creatinine) dysfunction. When plasma samples of septic mice exposed to the FAK-PyK2 inhibitor were evaluated, the concentrations of the same biomarkers were found at levels similar to those detected in the sham group (49.04 ± 5.49; 20.48 ± 1.37; 12.3 ± 0.86; 0.33 ± 0.05, respectively) (**Figures 2A–D**). Additionally, a slight increase in plasma urea levels was recorded in septic mice (81.65 ± 25.6), when compared to Sham animals (53.48 ± 4.6), but not in the CLP+PF271 group (35.5 ± 8.7) (**Figure 2E**).

### PF271 Administration Counteracts the Cytokine Storm Evoked by Experimental Sepsis

**Figure 3:**
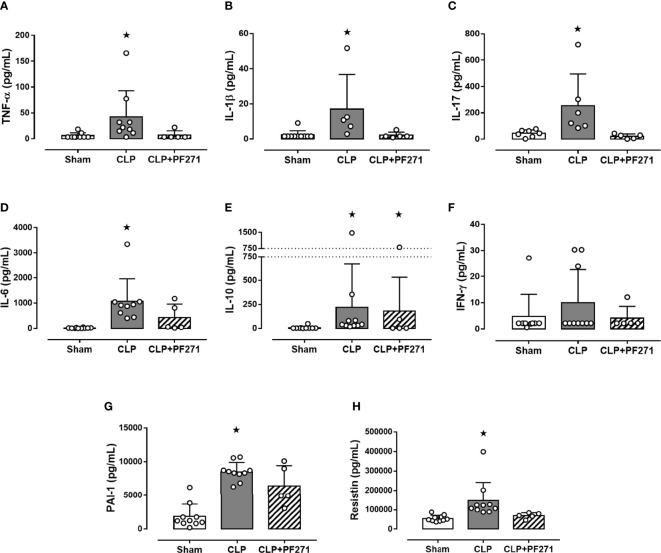
Effect of PF271 on plasma cytokines/hormones during experimental sepsis. Mice were randomly selected to undergo Sham or CLP surgery. One hour later, CLP mice were treated once with either Vehicle or PF271 (25 mg/kg s.c.). Twenty-four hours after Sham or CLP procedure, the animals’ blood was collected. Plasma levels of TNF-α **(A)**, IL-1β **(B)**, IL-17 **(C)**, IL-6 **(D)** IL-10 **(E)**, IFN-γ **(F)**, PAI-1 **(G)** and Resistin **(H)** were determined. Data are expressed as dot plots for each animal and as mean ± SD of 5-10 mice per group. TNF-α, IL-17, IL-6, IL-10 and Resistin and were statistically analyzed by one-way ANOVA followed by Bonferroni’s *post hoc*-test, while IL-1β, IFN-γ and PAI-1 were analyzed by Kruskal-Wallis followed by Dunn’s *post hoc*-test. *p < 0.05 CLP and/or CLP+PF271 *vs* Sham.

Twenty-four h after surgery, septic control mice developed a systemic cytokine storm, with a massive increase in the levels of pro-inflammatory cytokines, specifically TNF-α, IL-1β, IL-17, IL-6, and the proteins PAI-1 and Resistin, when compared to Sham animals. Most notably, treatment with PF271 completely abolished the CLP-induced increase in these markers (**Figures 3A–D, G, H**). Interestingly, sepsis also evoked a robust increase in plasma level of the anti-inflammatory cytokine IL-10, whose systemic concentration was not significantly affected by PF271 treatment (**Figure 3E**). On the contrary, neither the intervention (CLP) nor the PF271treatment significantly affected the systemic levels of IFN-γ (**Figure 3F**).

### PF271 Reverses Local Neutrophil Infiltration and Inflammation Evoked by Sepsis

**Figure 4:**
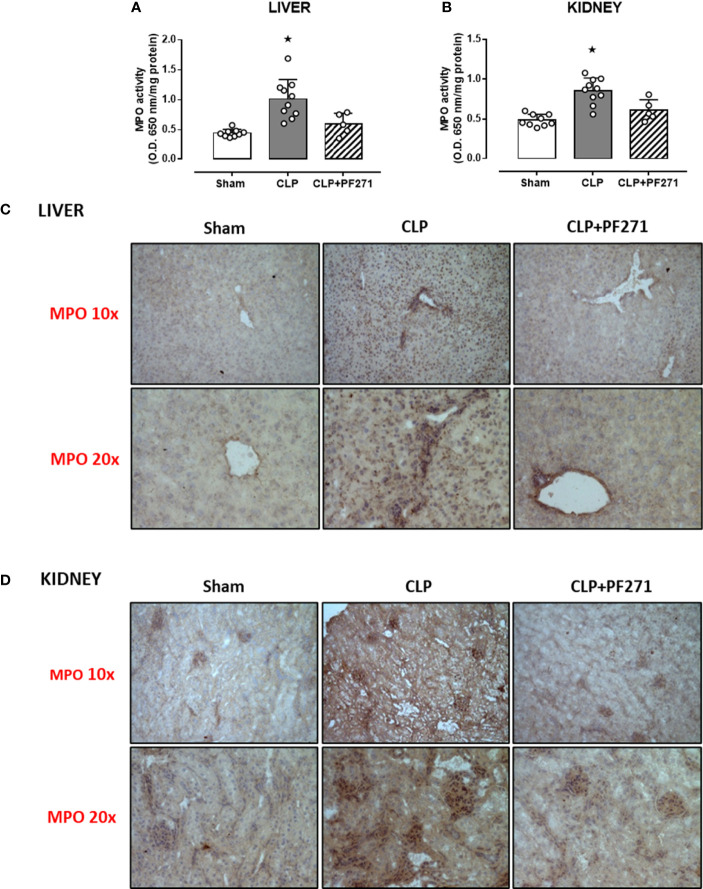
Effect of PF271 on neutrophil tissue infiltration during experimental sepsis. Mice were randomly selected to undergo Sham or CLP surgery. One hour later, CLP mice were treated once with either Vehicle or PF271 (25 mg/kg s.c.). Twenty-four hours after Sham or CLP procedure, liver and kidney were harvested. Through an *in vitro* assay, myeloperoxidase (MPO) activity was measured in liver **(A)** and kidney **(B)**. Tissue sections were prepared to identify MPO through immunohistochemistry assay in liver **(C)** and kidney **(D)**. Representative photomicrographs at 10x and 20x magnification were recorded of 5 animals per group. Data are expressed as dot plots for each animal and as mean ± SD of 5-10 mice per group for MPO activity. Statistical analysis was performed by one-way ANOVA followed by Bonferroni’s *post hoc*-test. *p < 0.05 CLP *vs* Sham/CLP+PF271.

**Figure 5:**
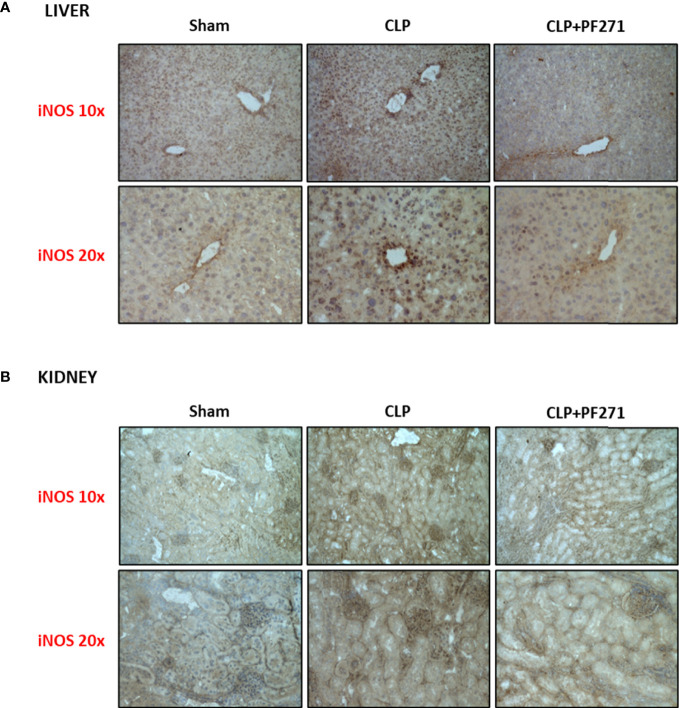
Effect of PF271 on tissue expression of iNOS during experimental sepsis. Mice were randomly selected to undergo Sham or CLP surgery. One hour later, CLP mice were treated once with either Vehicle or PF271 (25 mg/kg s.c.). Twenty-four hours after Sham or CLP procedure, liver and kidney were harvested. Tissue sections were prepared to identify iNOS expression through immunohistochemistry assay in liver **(A)** and kidney **(B)**. Representative photomicrographs at 10x and 20x magnification were recorded of 5 animals per group.

**Figure 6:**
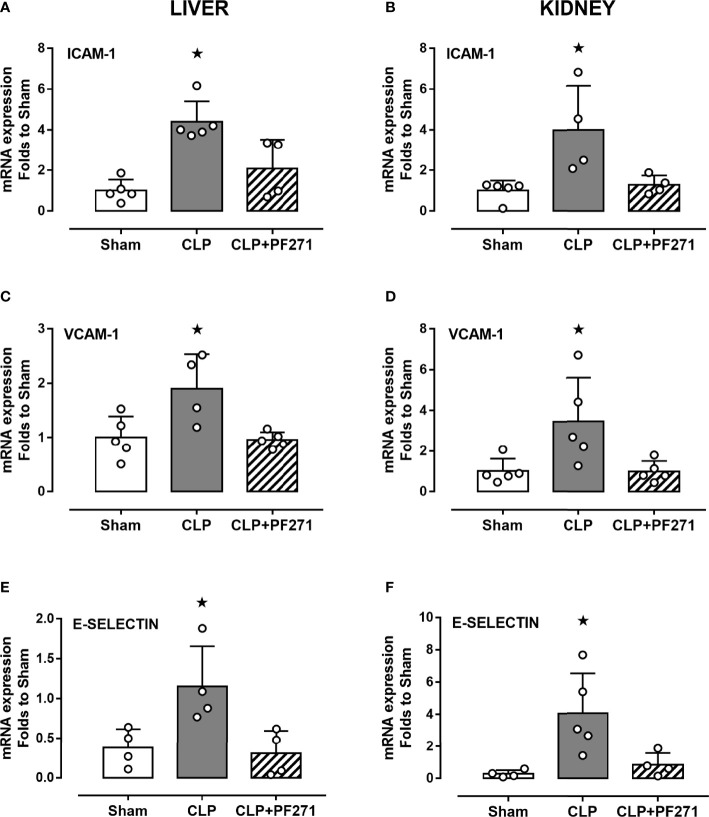
Effect of PF271 on tissue expression of adhesion molecules during experimental sepsis. Mice were randomly selected to undergo Sham or CLP surgery. One hour later, CLP mice were treated once with either Vehicle or PF271 (25 mg/kg s.c.). Twenty-four hours after Sham or CLP procedure, liver and kidney were harvested, and the total mRNA was extracted from them. Real time qPCR was performed for the following genes: ICAM-1 in liver **(A)** and kidney **(B)** extractions; VCAM-1 in liver **(C)** and kidney **(D)** extractions; E-Selectin in liver **(E)** and kidney **(F)** extractions. Relative gene expression was obtained after normalization to housekeeping genes (β-actin and GAPDH) using the formula 2^-ΔΔCT^ and folds change was determined by comparison to Sham group. Data are expressed as dot plots for each animal and as mean ± SD of 4-5 mice per group. Statistical analysis was performed by one-way ANOVA followed by Bonferroni’s *post hoc* test. *p < 0.05 CLP *vs* Sham/CLP+PF271.

As PF271 displayed protective effects against CLP-induced liver and kidney dysfunctions, we next investigated the possible underlying mechanisms. CLP injury doubled MPO activity in liver and kidney when compared to Sham mice, suggesting an increase in local polymorphonuclear cells (mainly neutrophils) infiltration. In contrast, the degree of MPO activity in CLP-mice treated with PF271 was similar to those recorded in sham mice (**Figures 4A, B**). The dramatic increase in MPO activity in liver (0.445 ± 0.02 *vs* 1.015 ± 0.10) and kidney (0.487 ± 0.02 *vs* 0.855 ± 0.05) following CLP injury was confirmed by immunohistochemistry analysis. While MPO immunopositivity was widely detected in the liver (**Figure 4C**) the increase in staining for MPO in the kidney was restricted to the renal corpuscles (**Figure 4D**). Interestingly, in both organs, treatment with PF271 abolished the increase in MPO-staining induced by experimental sepsis (**Figures 4C, D**). The increase in MPO-activity/staining seen in animals with CLP-sepsis and the prevention of this effect by PF271 treatment were mirrored by similar effects of CLP and PF271 on iNOS expression. As shown in **Figure 5**, iNOS immunopositivity was found to be increased in both liver (panel A) and kidney (panel B) of septic mice. Specifically, a high and wide expression/distribution of iNOS was observed in liver, being predominant in the periphery of blood vessels, whereas renal iNOS immunopositivity was increased mainly in the renal corpuscles and in the cortical tubules. Interestingly, PF271 treatment resulted in substantial reduction of the iNOS expression in both liver and kidney of septic mice (**Figures 5A, B**). The impact of the septic insult and the drug treatment on local inflammation was also confirmed by the gene-expression analysis of the adhesion molecules ICAM-1, VCAM-1 and E-Selectin, whose levels were drastically increased following the sepsis injury and significantly reduced by PF271 administration (**Figures 6A–F**).

### PF271 Reduces Local FAK-PyK2 Activity in Septic Mice

**Figure 7:**
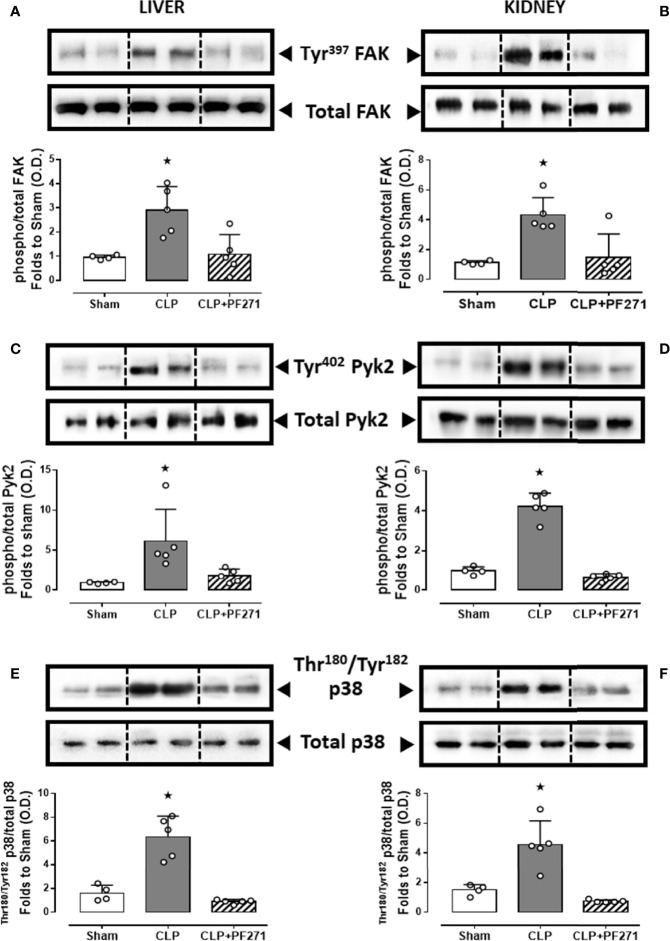
Effect of PF271 on tissue activation of FAK-PyK2 pathway during experimental sepsis. Mice were randomly selected to undergo Sham or CLP surgery. One hour later, CLP mice were treated once with either Vehicle or PF271 (25 mg/kg s.c.). Twenty-four hours after Sham or CLP procedure, liver and kidney were harvested, and the total protein was extracted from them. Western blotting analysis for phosphorylation of Tyr^397^ on FAK in the liver **(A)** and kidney **(B)** were normalized to total FAK; Phosphorylation of Tyr^402^ on PyK2 in the liver **(C)** and kidney **(D)** were normalized to total PyK2; Phosphorylation of Thr^180^/Tyr^182^ on p38 in the liver **(E)** and kidney **(F)** were normalized to total p38. Densitometric analysis of the bands is expressed as relative optical density (O.D.). Data are expressed as dot plots for each animal and as mean ± SD of 4-5 mice per group. Statistical analysis was performed by one-way ANOVA followed by Bonferroni’s *post hoc* test. *p < 0.05 CLP *vs* Sham/CLP+PF271.

To demonstrate that the above-mentioned effects were correlated with modulation of the pharmacological targets, we evaluated the local activation of FAK-PyK2 pathway. As shown in **Figure 7**, in liver and kidney both the septic insult and the drug treatment did not significantly modify the total expression of the FAK and PyK2 proteins. However, tissue homogenates of mice that underwent CLP exhibited a significant increase in both phosphorylation of Tyr^397^ on FAK (panels A-B) and Tyr^402^ on PyK2 (panels C-D), suggestive of increased enzyme activation. Furthermore, an overactivation of the downstream p38 MAPK protein triggered by FAK-PyK2 was also documented, as shown by a robust increase in the phosphorylation of Thr^180^/Tyr^182^ on p38 MAPK in liver (**Figure 7E**) and kidney (**Figure 7F**) of septic mice. As expected, mice treated with PF271 showed reduced activation of either FAK-PyK2 and its downstream effector p38 MAPK, confirming the ability of PF271 to interfere with its pharmacological targets in our experimental conditions (**Figures 7A–F**).

### NLRP3 Inflammasome Expression and Activity Are Attenuated by FAK-PyK2 Inhibition During Polymicrobial Sepsis

**Figure 8:**
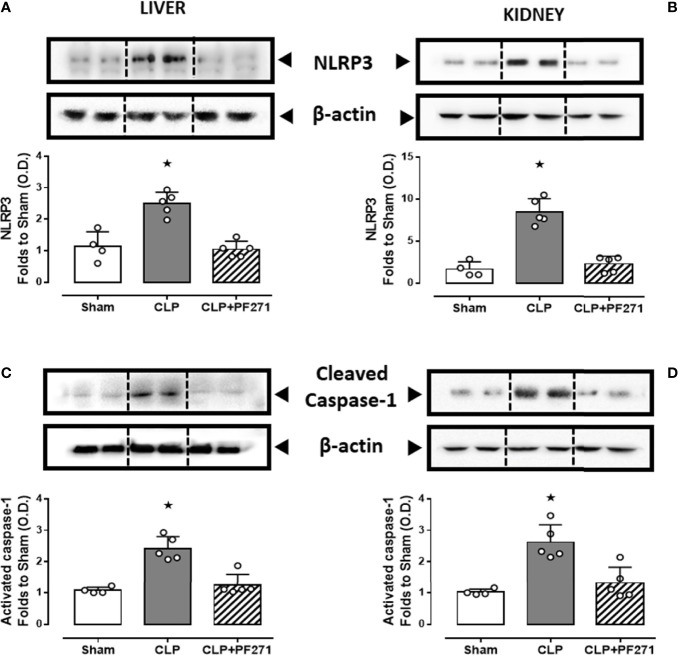
Effect of PF271 on tissue activation of the NLRP3 inflammasome during experimental sepsis. Mice were randomly selected to undergo Sham or CLP surgery. One hour later, CLP mice were treated once with either Vehicle or PF271 (25 mg/kg s.c.). Twenty-four hours after Sham or CLP procedure, liver and kidney were harvested, and the total protein was extracted from them. Western blotting analysis for NLRP3 in the liver **(A)** and kidney **(B)** were corrected against β-actin and normalized using the Sham related bands; Cleaved caspase-1 in the liver **(C)** and kidney **(D)** were corrected against β-actin and normalized using the Sham related bands. Densitometric analysis of the bands is expressed as relative optical density (O.D.). Data are expressed as dot plots for each animal and as mean ± SD of 4-5 mice per group. Statistical analysis was performed by one-way ANOVA followed by Bonferroni’s *post hoc* test. *p < 0.05 CLP *vs* Sham/CLP+PF271.

We and others (33, 38, 39) have previously documented a pivotal role of the NLRP3 inflammasome pathway in mediating deleterious effects in sepsis. We, therefore, explored a potential cross-talk mechanism linking FAK-PyK2 inhibition to impaired NLRP3 activity. As shown in **Figure 8**, experimental sepsis evoked a robust increase in the assembly of the NLRP3 complex in liver and kidney, which was associated with a subsequent increase in the cleavage of caspase-1, when compared to animals in the Sham group. In contrast, mice subjected to CLP and treated with PF271 showed a significant reduction in the renal and hepatic expression and activation of the NLRP3 inflammasome complex.

## Discussion

Preliminary *in vitro* and *in vivo* studies have previously suggested a role of the FAK-Pyk2 pathways in inflammation and, ultimately, organ damage caused by LPS (23, 24, 40, 41). Here we show, for the first time, that administration of PF271, a selective dual inhibitor of both FAK and Pyk2, ameliorates the severity score and prolongs the survival in a murine model of CLP-sepsis. Mice subjected to sepsis (and treated with vehicle) developed local and systemic inflammation, severe organ injury/dysfunction and this was associated with a high mortality. In contrast, administration of the reversible inhibitor for both FAK and Pyk2, PF271, counteracted all these abnormalities caused by sepsis and resulted in long-term protection and improved survival. In this study, we confirmed that sepsis does, indeed, lead to phosphorylation and, hence, activation of both FAK and Pyk2 and we show that PF271 attenuates FAK-Pyk2 phosphorylation, and thus activation, in septic mice. In addition, we explored the molecular mechanisms involved in the deleterious effects attributed to FAK-Pyk2 overactivation during sepsis. In the liver and kidney of septic mice, we documented that PF271 attenuated the upregulation of the cellular adhesion molecules (CAMs) VCAM-1, ICAM-1 and E-Selectin, which exert a pivotal role in driving leukocyte infiltration and the following excessive inflammatory response, which ultimately lead to the development of MOF (42–44). We also documented that PF271 administration was associated with a significant reduction in local (liver and kidney) expression and activity of either MPO, a well-known biomarker of neutrophil infiltration (45), and iNOS, which is detectable in neutrophils, leading to overproduction of nitric oxide (NO) and the following generation of other reactive species (46). Our study does not allow the identification of the specific cell types involved in PF271-mediated responses. Nevertheless, it is well described that cells of the innate immune system and endothelial cells are the most prominent type involved in the exorbitant release of inflammatory cytokines, which in turn drives septic inflammation (47) and the same cells express the FAK-PyK2 cascade, whose role in the cellular production of inflammatory mediators has been clearly demonstrated (17, 22, 40, 48–50). We may, thus, speculate that the beneficial effects of PF271, including those related to the preservation of organ function in sepsis, are due, at least in part, to a direct effect of PF271 on both leukocytes and endothelial cells.

One of the most relevant downstream signaling events directly activated by FAK-Pyk2 is the MAPK p38-dependent signaling pathway, which in turn activates the NF-κB transcription factor, leading to cytokines overproduction and, thus, the cytokine storm typical of the systemic inflammation and organ dysfunction associated to sepsis (17, 40, 50, 51). Here we confirmed a local overactivation of the p38 MAPK signaling during experimental sepsis, which was significantly reduced by PF271 treatment. The sepsis-induced activation of p38 MAPK was paralleled by a massive increase in the systemic levels of proinflammatory cytokines, such as TNF-α, IL-1β, IL-17 and IL-6, as well as the anti-inflammatory cytokine IL-10. Most notably, we report that all pro-inflammatory cytokines had an impressive reduction when mice were treated with PF271, whereas high levels of the anti-inflammatory cytokine IL-10 were unaffected by treatment. In keeping with clinical studies (52, 53), we also documented a significant sepsis-induced upregulation of the recently discovered inflammatory cytokine resistin and, most notably, we demonstrated here, for the first time, that its systemic concentrations may be affected by the pharmacological modulation of the FAK-Pyk2 pathway. The reduction in resistin levels caused by PF271 in sepsis could contribute, at least in part, to the prolonged survival of septic mice following drug treatment, as recent findings have demonstrated that resistin significantly impairs neutrophil killing of Gram-positive and Gram-negative organisms, thus contributing to the development of immunosuppression, which is central to sepsis-related morbidity and mortality (54). However, further studies are needed to support this hypothesis. One of the cytokines showing a relevant modulation by either sepsis and PF271 was IL-1β. IL-1β is primarily released from the activated NLRP3 inflammasome, which plays a pivotal role in the pathogenesis of sepsis (55). We thus evaluated the potential impact of the FAK-Pyk2 pharmacological modulation on the NLRP3 complex formation and activation showing that the treatment with PF271 abolished the NLRP3 overexpression and caspase-1 overactivation secondary to sepsis, which was paralleled by systemic reduction in IL-1β. Our data strengthen previously published experimental data demonstrating that Pyk2 directly phosphorylates monomeric apoptosis-associated speck-like protein containing CARD (ASC) subunits of the NLRP3 inflammasome, bringing ASC into an oligomerization-competent state to then activate caspase-1 and trigger IL-1β secretion (21).

During experimental sepsis, we also documented a significant overproduction of plasminogen activator inhibitor-1 (PAI-1), the serum levels of which have been reported to rapidly increase in the early stages of sepsis and are positively correlated to sepsis severity in humans (56). PAI-1 overproduction may also contribute to the FAK activation observed in our experimental model as PAI-1 has been demonstrated to induce FAK phosphorylation leading to processes of macrophage infiltration (57). The slight reduction in PAI-1 levels observed in septic mice following PF271 administration could be due, at least in part, to the reduced systemic concentrations of cytokines, mainly IL-6, which has been reported to exert a key role in regulating PAI-1 expression in vascular endothelial cells (58). We also found high plasmatic levels of lactate, a marker of tissue perfusion, during experimental sepsis and we reported the treatment with PF271 brought its levels to values not different from the control group. Plasma lactate is not only a marker of tissue perfusion, but it is currently used as a diagnostic criterion to determine whether a patient is in septic shock (1). Thus, our findings also demonstrate the ability of PF271 to improve hemodynamic factors during sepsis. However, we must acknowledge that the lack of direct measurements of hemodynamic parameters does not allow us to reach a solid conclusion on this specific issue.

Our study has further limitations, including the lack of evidence of any effects of PF271 on other important organs involved in MOF associated with sepsis, such as the lungs and the heart, as well as the use of only male mice, which does not allow to get a better understanding of the role of gender dimorphism in the effects of PF271. Although the experimental procedures reported here are in keeping with most of the recommendations reported in the MQTiPSS consensus guidelines (29), we did not consider the use of repeated analgesic and antimicrobial treatments as well as continuous fluid resuscitation. Furthermore, all efficacy endpoints that we evaluated, and the overall survival were assessed only at day 1 and day 5 post-CLP, respectively, and multiple and longer kinetics are needed for a better understanding of the overall efficacy of PF271 on the dynamic changes taking place in sepsis. Thus, future studies are required to improve the clinical relevance of our findings as well as to gain a better insight into the safety profile of the proposed drug treatment.

## Conclusions

This study shows for the first time that the development of organ dysfunction induced by a clinically relevant polymicrobial sepsis model is associated with activation of the FAK-Pyk2 pathway, while the pharmacological inhibition of this pathway results in protective effects and prolonged survival. This beneficial effect is likely to be due to the prevention of leukocyte infiltration and related excessive local inflammation through the modulation of FAK-Pyk2 downstream inflammatory cascades, including p38-MAPK and NLRP3 inflammasome. As PF271 has already been tested in a phase I clinical trial in oncology, our finding may provide an opportunity for the repurposing of this compound for the use in patients with sepsis.

## Data Availability Statement

The raw data supporting the conclusions of this article will be made available by the authors, without undue reservation.

## Ethics Statement

The animal study was reviewed and approved by Ministero della Salute, UFFICIO VI, Tutela del benessere animale, igiene zootecnica e igiene urbana veterinaria.

## Author Contributions

GA, DF, and MC conceived and designed the experiments. GA, EA, GE, RM, JG, DC, and EP performed the experiments. GA, EA, RM, JG, MA, CC, RS, CT, DF, and MC analyzed the data. GA, MA, CC, RS, CT, DF, and MC contributed to the writing of the manuscript. All authors reviewed the manuscript before submission.

## Funding

This work was supported and funded by the Università degli Studi di Torino (Ricerca Locale 2020 and 2021) and by the Foundation for Research and Innovation of the State of Santa Catarina (2021TR000318).

## Conflict of Interest

The authors declare that the research was conducted in the absence of any commercial or financial relationships that could be construed as a potential conflict of interest.

## Publisher’s Note

All claims expressed in this article are solely those of the authors and do not necessarily represent those of their affiliated organizations, or those of the publisher, the editors and the reviewers. Any product that may be evaluated in this article, or claim that may be made by its manufacturer, is not guaranteed or endorsed by the publisher.
