# Supplementary material for: Pharmacological Inhibition of FAK-Pyk2 Pathway Protects Against Organ Damage and Prolongs the Survival of Septic Mice
Source: Front Immunol. 2022 Feb 1;13:837180. doi: 10.3389/fimmu.2022.837180 (PMC8843946; doi:10.3389/fimmu.2022.837180)
Supplement: Supplementary file 1 [file DataSheet_1.docx]

Supplementary Material

**CLINICAL SCORE PROTOCOL**

To assess the animals' well-being, every 24 hours after surgery, a severity score was applied. Using a modified murine sepsis score (MSS) [1], we evaluated it through the observation of six components: lethargy, piloerection, tremors, periorbital exudates, respiratory distress and diarrhoea [2]. The score was given by the presence or absence of each of the six variables. Each variable was positive when the animal had one or more items described in the second column of table 1.

An additional criterion for humane euthanasia was applied to mice under survival study: mice with high clinical score (= 6) associated with progressive weight loss exceeding 20% of baseline weight. No mice reached this criterion during our study.

**Table 1. Modified Murine Sepsis Score to assess the severity of disease in CLP-induced sepsis**

| **Variable** | **Description** |
| --- | --- |
| - Lethargy | - Mouse shows impairment in any of the following: eating, drinking, climbing, running, fighting - Mouse only moves when provoked - Slow or absent movements - No response to auditory stimulus |
| - Piloerection | - Patches of fur piloerected - Majority of back is piloerected |
| - Tremors | - Tremors when moving or climbing |
| - Periorbital exudates | - Eyes not fully open, possibly with secretions - Eyes at least half closed, possibly with secretions - Eyes fully closed, possibly with secretions |
| - Respiratory distress | - Brief periods of laboured breathing - Laboured with intermittent gasps - Gasping |
| - Diarrhoea | - Presence of liquid stools |

**MINIMUM QUALITY THRESHOLD IN PRE-CLINICAL SEPSIS STUDIES (MQTiPSS) CONSENSUS GUIDELINES RECOMMENDATIONS [3][4][5][6]**

**Elements followed as recommended in MQTiPSS consensus guidelines:**

- Polymicrobial sepsis model with injury initiated by the peritoneal cavity
- Sepsis model with proof of organ dysfunction
- Survival follow-up reflects the clinical course of the sepsis model
- Therapeutic intervention starts after the septic insult replicating clinical care
- Randomized Treatment
- Methodology provides information enough to enable replication, being in accordance with ARRIVE guidelines
- Experiments are performed on mammals, middle-aged
- Standardized criteria to monitor the well-being of septic animals (clinical score, body temperature and body weight)
- Standardized criteria for euthanasia of septic animals
- Administration of analgesic for surgical sepsis consistent with ethical considerations
- Single administration of fluid resuscitation after surgical procedure (saline solution, prewarmed 37° C)
- Fully explored animal model

**Elements not followed as recommended in MQTiPSS consensus guidelines:**

- Blinded treatment
- Development of a scoring system to define objective evidence of the severity of organ/system dysfunction
- Continuous administration of fluid resuscitation/analgesic
- Administration of antimicrobials

**REFERENCES**

1. Shrum, B., Anantha, R. V., Xu, S. X., Donnelly, M., Haeryfar, S. M., McCormick, J. K., & Mele, T. (2014). A robust scoring system to evaluate sepsis severity in an animal model. BMC research notes, 7(1), 1-11. doi: 10.1186/1756-0500-7-233
2. O'Riordan, C. E., Purvis, G. S., Collotta, D., Krieg, N., Wissuwa, B., Sheikh, M. H., ... & Thiemermann, C. (2020). X-linked immunodeficient mice with no functional Bruton's Tyrosine Kinase are protected from sepsis-induced multiple organ failure. Frontiers in immunology, 11. doi: 10.3389/fimmu.2020.581758
3. Osuchowski, M. F., Ayala, A., Bahrami, S., Bauer, M., Boros, M., Cavaillon, J. M., ... & Zingarelli, B. (2018). Minimum quality threshold in pre-clinical sepsis studies (MQTiPSS): an international expert consensus initiative for improvement of animal modeling in sepsis. Intensive care medicine experimental, 6(1), 1-6. doi: 10.1097/SHK.0000000000001212
4. Zingarelli, B., Coopersmith, C. M., Drechsler, S., Efron, P., Marshall, J. C., Moldawer, L., ... & Thiemermann, C. (2019). Part I: Minimum Quality Threshold in Pre-Clinical Sepsis Studies (MQTiPSS) for Study Design and Humane Modeling Endpoints. Shock (Augusta, Ga.), 51(1), 10. doi: 10.1097/SHK.0000000000001243
5. Libert, C., Ayala, A., Bauer, M., Cavaillon, J. M., Deutschman, C., Frostell, C., ... & Remick, D. G. (2019). Part II: Minimum Quality Threshold in Pre-Clinical Sepsis Studies (MQTiPSS) for Types of Infections and Organ Dysfunction Endpoints. Shock (Augusta, Ga.), 51(1), 23. doi: 10.1097/SHK.0000000000001242
6. Hellman, J., Bahrami, S., Boros, M., Chaudry, I. H., Fritsch, G., Gozdzik, W., ... & Huber-Lang, M. (2019). Part III: minimum quality threshold in preclinical sepsis studies (MQTiPSS) for fluid resuscitation and antimicrobial therapy endpoints. Shock, 51(1), 33-43. doi: 10.1097/SHK.0000000000001209
